# Ambulatory antibiotic prescription rates for acute respiratory infection rebound two years after the start of the COVID-19 pandemic

**DOI:** 10.1371/journal.pone.0306195

**Published:** 2024-06-25

**Authors:** Elizabeth R. Stevens, David Feldstein, Simon Jones, Chelsea Twan, Xingwei Cui, Rachel Hess, Eun Ji Kim, Safiya Richardson, Fatima M. Malik, Sumaiya Tasneem, Natalie Henning, Lynn Xu, Devin M. Mann

**Affiliations:** 1 Department of Population Health, NYU Grossman School of Medicine, New York, New York, United States of America; 2 Department of Medicine, University of Wisconsin School of Medicine and Public Health, Madison, Wisconsin, United States of America; 3 Department of Population Health Sciences, University of Utah, Salt Lake City, Utah, United States of America; 4 Northwell, New Hyde Park, New York, United States of America; 5 Medical Center Information Technology, NYU Langone Health, New York, New York, United States of America; National Center for Global Health and Medicine, JAPAN

## Abstract

**Background:**

During the COVID-19 pandemic, acute respiratory infection (ARI) antibiotic prescribing in ambulatory care markedly decreased. It is unclear if antibiotic prescription rates will remain lowered.

**Methods:**

We used trend analyses of antibiotics prescribed during and after the first wave of COVID-19 to determine whether ARI antibiotic prescribing rates in ambulatory care have remained suppressed compared to pre-COVID-19 levels. Retrospective data was used from patients with ARI or UTI diagnosis code(s) for their encounter from 298 primary care and 66 urgent care practices within four academic health systems in New York, Wisconsin, and Utah between January 2017 and June 2022. The primary measures included antibiotic prescriptions per 100 non-COVID ARI encounters, encounter volume, prescribing trends, and change from expected trend.

**Results:**

At baseline, during and after the first wave, the overall ARI antibiotic prescribing rates were 54.7, 38.5, and 54.7 prescriptions per 100 encounters, respectively. ARI antibiotic prescription rates saw a statistically significant decline after COVID-19 onset (step change -15.2, 95% CI: -19.6 to -4.8). During the first wave, encounter volume decreased 29.4% and, after the first wave, remained decreased by 188%. After the first wave, ARI antibiotic prescription rates were no longer significantly suppressed from baseline (step change 0.01, 95% CI: -6.3 to 6.2). There was no significant difference between UTI antibiotic prescription rates at baseline versus the end of the observation period.

**Conclusions:**

The decline in ARI antibiotic prescribing observed after the onset of COVID-19 was temporary, not mirrored in UTI antibiotic prescribing, and does not represent a long-term change in clinician prescribing behaviors. During a period of heightened awareness of a viral cause of ARI, a substantial and clinically meaningful decrease in clinician antibiotic prescribing was observed. Future efforts in antibiotic stewardship may benefit from continued study of factors leading to this reduction and rebound in prescribing rates.

## Introduction

For decades antibiotic prescribing rates in the United States have remained persistently high. Despite efforts to educate patients and clinicians regarding the ineffectiveness of antibiotics in most acute respiratory infections (ARIs), prescribing rates have remained relatively stable since the late 1990’s and use of broad spectrum antibiotics has actually increased in some instances [1, 2]. Furthermore, it is estimated that 50% of the outpatient ARI antibiotic prescriptions are inappropriate [3, 4]. However, in 2020, coinciding with the rise of the COVID-19 pandemic, a rapid decline in antibiotic prescription rates was observed in ambulatory care settings [5–9].

After an initial spike in use at the start of 2020, antibiotic prescribing in ambulatory care settings decreased by 40–80% compared to the same time in prior years [5–9]. By early 2021, most antibiotic prescribing had still not rebounded to pre-COVID-19 levels [5–7]. The exact factors driving the decline in antibiotic prescribing rates are still unknown, but have been hypothesized to be connected to a decline in overall seasonal respiratory infection rates, [5] as well as changes in patient and clinician behaviors [6–8]. Regardless, it is still uncertain whether the impact of COVID-19 on antibiotic prescribing is temporary or if these represent a lasting change in the trajectory of antibiotic prescribing rates.

If maintained, the observed decline in antibiotic prescribing has important implications for efforts targeting antibiotic stewardship. However, it is currently unclear if antibiotic prescription rates will remain lowered as health systems emerge from the COVID-19 pandemic and more patients return to using ambulatory care. Now more than two years after the initial observed decline, we sought to determine whether antibiotic prescribing rates for ARI in ambulatory care have remained suppressed compared to pre-COVID-19 levels. We performed a trend analysis of antibiotics prescribed for ARI in ambulatory care practices within four academic health systems in New York, Wisconsin, and Utah between January 2017 and June 2022. We compared this trend to prescribing for Urinary Tract Infections (UTIs) over the same period.

## Methods

### Data and setting

Ambulatory care data was retrospectively collected from the electronic health records (EHR) of four large academic health care systems in New York (two sites, henceforth referred to as NY-A and NY-B), Utah, and Wisconsin between the period of January 1, 2017 and June 30, 2022 (December 31, 2021 for NY-B and Wisconsin). Data were collected on February 8, 2023 from 255 primary care practices and 68 urgent care practices within the four health systems (S1 Table). All health system primary and urgent care practices that use the health system EHR were included in the analyses.

All patient encounters occurring within the timeframe of interest at the primary care and urgent care practices were included in the analyses if they were an ARI or UTI ambulatory care visit. An ARI or UTI ambulatory care visit was defined as any outpatient office, telemedicine, or urgent care visit with an ARI or UTI ICD-10 code listed as a diagnosis, respectively (see Supplement S2 Table for included ICD-10 codes). ICD-10 codes were converted from ICD-9 codes previously used by Linder et al. 2009 [10]. UTI was included as a comparator to represent a similarly common ambulatory prescribed antibiotic not clinically connected to COVID-19 and could serve as useful comparison to ARI antibiotic prescribing. An ARI or UTI antibiotic prescription was defined as a distinct encounter with an antibiotic order associated with the encounter within 7 days of a completed ARI or UTI visit (See supplement S2 Table for included antibiotic types). All study procedures were performed in accordance with the Declaration of Helsinki. All protocol procedures were approved by the NYU Langone Health Institutional Review Board (IRB). Informed consent was waived for the use of EHR data by the NYU Langone Health IRB. Data was de-identified for analyses.

### Statistical analysis

In an effort to understand the likely impact of the COVID-19 pandemic on ARI and UTI antibiotic prescription rates, we used an interrupted time series (ITS) model. This approach is a quasi-experimental design that has been extensively used to evaluate the impact of policy changes, interventions, and other events. ITS models explain the impact of an event in terms of change in level as well as changes in trend [11]. Model results were reported as the antibiotic prescribing rate slope change from baseline (pre-COVID-19) for the periods of COVID-19 first wave and post-COVID-19 first wave. The first wave was defined as the time period when the COVID-19 pandemic cases began to rise (end of March 2020) until COVID-19 rates reached their first trough (end of May 2021). Total change in antibiotic prescribing rates are presented as a step change of prescriptions per 100 non-COVID ARI encounters compared to the estimated trend at the same time period if the COVID-19 pandemic had not occurred. The slope change and step change are measures signifying two different phenomena; the slope represents the rate of prescribing change over time, whereas the step change signifies the total change in prescribing that has occurred in that period of time in addition to the expected change based on slope trends.

Our first step was to examine the number of new COVID-19 cases per month to identify the beginning and end of the first wave using Change Point Analysis [12]. This approach can detect multiple changes in level of the number of new COVID-19 cases. COVID-19 positivity rates over time were calculated nationally and by state using surveillance data for New York, Utah, and Wisconsin using 7-day average reported positive cases divided by population [13–17]. Given that the impact of COVID-19 on antibiotic prescribing will be a mixture of both reaction to organizational policies, national news, and the pandemic’s local impact on the population and health care systems, we first applied a Change Point Analysis to the monthly data used for the entire USA (See supplement S1 Fig for results of COVID-19 first wave Change Point Analysis). The national change points where then compared to the local data for each state to see if they needed to be adjusted. Because incidence of ARI diagnoses vary by season, [18] we removed seasonality from ARI antibiotic prescribing rates using multiplicative seasonal decomposition by applying the algorithm described in Kendell and Stewart [19]. This allowed for the comparison of overall trends in ARI throughout the year, including comparisons between times with high seasonality that would otherwise be incomparable. For all analyses the April 2020 observation was excluded from ARI and UTI models as an outlier due to low visit volume.

Finally, we conducted an ITS on the de-seasonalized antibiotic prescription rate data with the interruptions supplied from the change point analysis. Analyses were performed for each site individually followed by a combined multi-site analysis. The ITS was estimated using R’s NLME [20] package which takes into account autocorrelation of a time series. The order of autocorrelation was estimated by examination of the autocorrelation and partial autocorrelation plots following the procedure outlined by Box et al 2016 [21]. Statistical significance was defined as a p<0.05. All analyses were carried out using R version 4.2.1.

## Results

**Fig 1:**
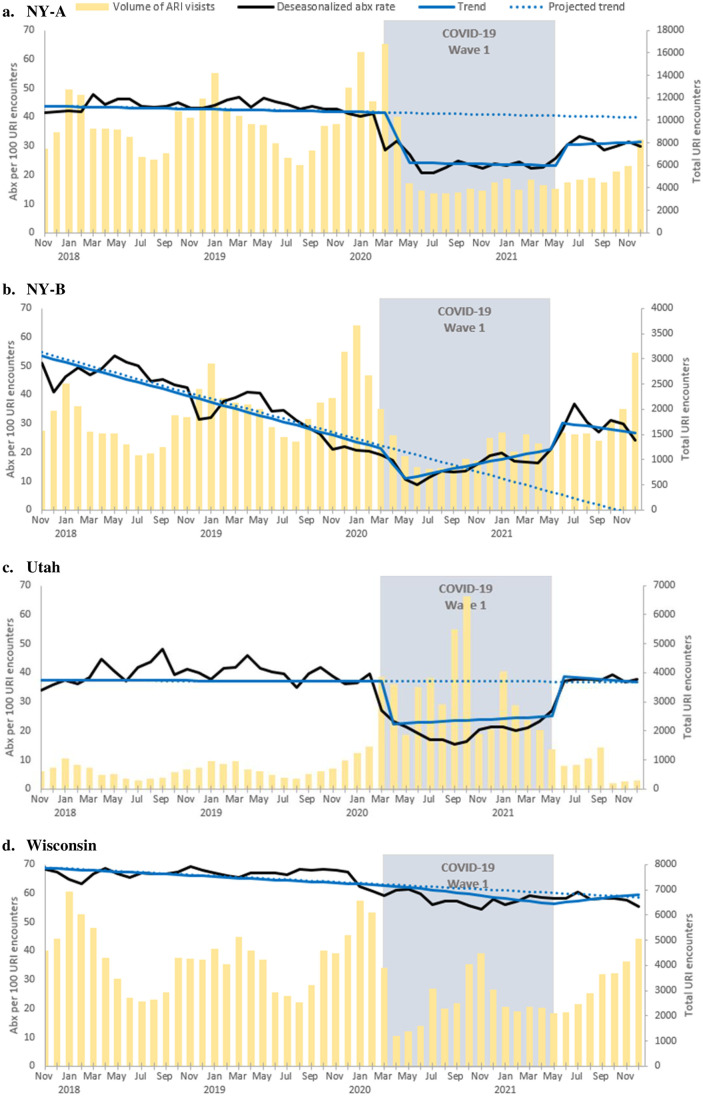
a-d. Rate of ARI encounters with antibiotic prescriptions by month, 2017–2022, by site.

**Fig 2:**
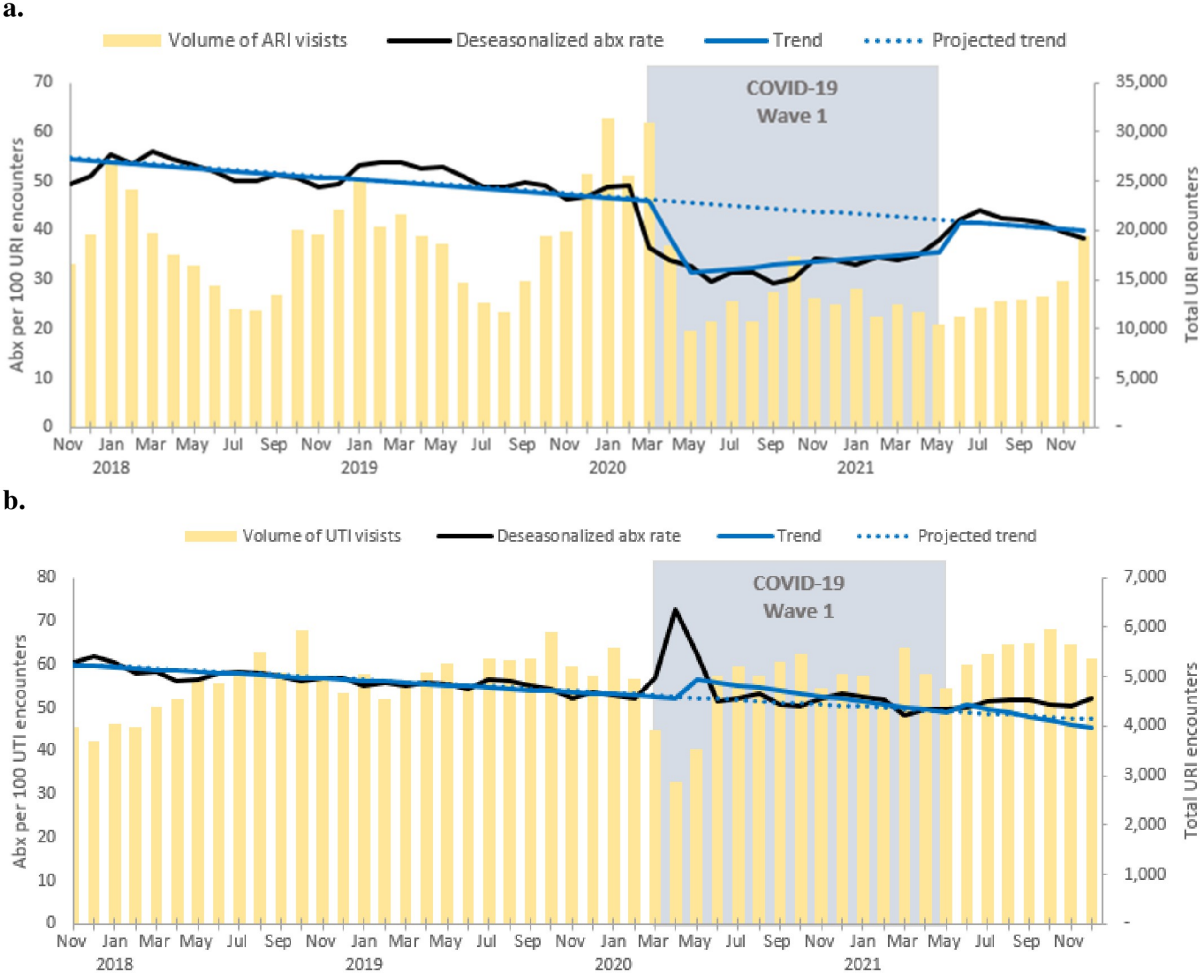
a, b. Aggregate rate of (a)ARI and (b) UTI encounters with antibiotic prescriptions by month, 2017–2022.

ARI antibiotic prescribing rates and trend over time are presented by site in Fig 1a–1d and in aggregate in Fig 2a. Slope and step change data for each study site can be found in Table 1. Examination of the autocorrelation and partial autocorrelation plots suggested that all-time series were autoregressive order one processes (AR1). Between January 1, 2017 and December 31, 2021 a total of 842,648 ARI encounters occurred, including 566,774 ARI encounters prior to COVID-19, 179,162 ARI encounters during the first wave, and 96,712 ARI encounters post the first wave. Prior to COVID-19, overall there was a small but significant negative trend in ARI antibiotic prescribing observed (slope -0.3, 95% CI: -0.5 to -0.1). Two sites had significant negative trends in ARI antibiotic prescribing rates: NY-B (slope -1.15, 95% CI: -1.43 to -0.86) and Wisconsin (slope -0.2, 95% CI: -0.4 to -0.01). At baseline the overall ARI antibiotic prescribing rate was 54.7 prescriptions per 100 encounters.

**Table 1:** ARI antibiotic prescribing rate trends in the periods of pre-COVID-19, COVID-19 first wave, and post-COVID-19 first wave.

	Overall		NY-A		NY-B		Utah		Wisconsin	
	Value	95% CI	Value	95% CI	Value	95% CI	Value	95% CI	Value	95% CI
**Intercept**	54.7	51.76 to 57.65	44.67	42.62 to 46.73	54.66	49.66 to 59.65	37.49	31.33 to 43.65	69	65.36 to 72.63
**Slope Pre-COVID-19**	-0.3	-0.47 to -0.13	-0.08	-0.17 to 0.01	-1.15	-1.43 to -0.86	-0.01	-0.26 to 0.24	-0.21	-0.42 to -0.01
**Step change first wave**	-15.16	-20.41 to -9.92	-17.17	-21.53 to -12.81	-12.22	-19.6 to -4.83	-14.96	-20.85 to -9.07	0.33	-3.91 to 4.57
**Slope change first wave**	0.62	0.05 to 1.19	0	-0.46 to 0.46	1.99	1.09 to 2.9	0.21	-0.64 to 1.07	-0.27	-0.87 to 0.33
**Step change post first wave**	0.01	-6.13 to 6.16	-10.18	-14.49 to -5.88	25.57	13.67 to 37.46	1.96	-9.3 to 13.22	-3.33	-10.7 to 4.05
**Slope change post first wave**	0.03	-0.32 to 0.39	0.23	-0.04 to 0.51	0.55	-1.24 to 2.34	-0.31	-0.93 to 0.31	0.68	0.26 to 1.09

ARI antibiotic prescriptions rates overall saw a significant decline after the initial onset of the COVID-19 pandemic (step change -15.2, 95% CI: -20.4 to -9.9). During the first wave of COVID-19 there was a small but significant positive change in the overall trend in ARI antibiotic prescribing (slope 0.6, 95% CI: 0.05 to 1.2), however variability existed across sites with NY-B (slope change 1.99, 95% CI: 1.09 to 2.90) having a significant positive slope change from baseline and no other sites having a significant slope change. During the first wave, the average overall ARI antibiotic prescribing rate was 39.5 prescriptions per 100 encounters. During the first wave, overall encounter volume (179,162 encounters) was decreased by 29.4% from baseline (253,875 encounters) from the same time period the year prior to COVID-19 onset (Fig 2a).

After the first wave, ARI antibiotic prescription rates overall were no longer significantly suppressed from baseline (step change 0.01, 95% CI: -6.1 to 6.2), however one site, NY-A (step change -10.18, 95% CI: -14.49 to -5.88) remained significantly suppressed from baseline and one site, NY-B, saw a significant increase in ARI antibiotic prescription rates (step change 25.57, 95% CI: 13.67 to 37.46). After the first wave of COVID-19 there was a significant positive change in the overall trend in ARI antibiotic prescribing compared to baseline (slope 0.87, 95% CI: 0.51 to 1.23). Only one of the sites, Wisconsin, saw a significant change with the slope increasing to 0.7 (95% CI: 0.3 to 1.1) as compared to baseline. After the first wave, the average overall ARI antibiotic prescribing rate was 54.7 prescriptions per 100 encounters. During the period after the first wave, overall encounter volume remained decreased by 18.8% (96,712 encounters) from baseline (119,067 encounters) during the same time period the year prior to COVID-19 onset.

Between January 1, 2017 and December 31, 2021 a total of 284,447 UTI encounters occurred. Overall, antibiotic prescribing rates for UTI saw a significant positive change after the onset of the COVID-19 pandemic (step change 5.2, 95% CI 0.2 to 10.1) (Fig 2b), which was maintained after the first wave. Prior to COVID-19 there was an overall significant negative trend in UTI antibiotic prescribing rate (slope -0.3, 95% CI -0.5 to -0.1) that was no longer significant after the first wave. NY-A, NY-B, and Wisconsin had significant negative trends in UTI antibiotic prescribing rates, which were no longer significant by the end of the first wave. Conversely, after the first wave, Utah’s significant positive trend for UTI antibiotic prescriptions had become non-significant. Disaggregated prescribing trends for each study site can be found in supplement S3-S6 Figs in S1 File. There was no significant difference between the UTI antibiotic prescriptions rate at baseline vs the end of the observation period. See supplement S3 Table for UTI slope and step changes.

## Discussion

This research represents one of the first multisite studies examining antibiotic prescribing trends for ARI in ambulatory care settings more than one year after the onset of the COVID-19 pandemic and the initial observed decline in ARI antibiotic prescribing. This study revealed a near complete rebound in ARI antibiotic prescribing rates to pre-COVID-19 levels by the end of 2021, as observed in another study using claims data [22]. These findings confirm the previous research that identified the initial drop in ARI antibiotic prescribing rates at the onset of COVID-19, [5–9] and builds upon those that detected a potential upward trend in antibiotic prescription rates early in 2021 [5–7]. The observed reduction of ARI antibiotic prescribing rates, but not for other non-respiratory conditions, such as UTI, is consistent with other studies on the impact of the COVID-19 pandemic on antibiotic prescribing trends [23].

While there was variation between study sites, the full rebound of antibiotic prescribing rates for ARI in ambulatory care settings emphasizes the continued need for increased antibiotic stewardship in these settings. In the US an estimated 50% of all outpatient antibiotic prescriptions for ARIs are inappropriate [3, 4]. From 1996–2010, 72% of adult primary care patients with a diagnosis of acute bronchitis received antibiotics contrary to guideline recommendations against antibiotic treatment and prescription rates actually increased during this time frame [24]. Patients with sore throats received antibiotics 61% of the time when the prevalence of Group A streptococcus, the only clear indication for antibiotics, is only 10% in adults [25]. While there were existing downward trends in antibiotic prescribing at some study sites prior to the pandemic, the step change in antibiotic prescribing rates at the onset of COVID-19 indicates the potential for further reductions in antibiotic prescribing. However, the return to pre-COVID-19 prescription rates, and likely pre-COVID-19 inappropriate prescribing, highlights the stubborn nature of the antibiotic overprescribing habit and emphasizes the need to develop innovative strategies to persuade prescribers of antimicrobials to follow evidence-based prescribing practices.

Although the results of this analysis cannot be used to draw causational conclusions as to why the observed changes in antibiotic prescribing occurred, the significant decrease and subsequent rebound in ARI antibiotic prescribing suggests that antibiotic prescribing rates are potentially modifiable. Understanding the unique environment created by the COVID-19 pandemic may hold clues for the development of effective antibiotic stewardship interventions. For example, previous research has shown that a substantial reason for prescribing antibiotics is the drive to meet patient expectations [23]. Therefore, as previously suggested, the observed reduction in antibiotic prescribing during the first wave of COVID-19 may be a result of changes in patient and clinician behaviors [6–8].

One hypothesis to explain the initial decline in ARI antibiotic prescriptions post-first wave of COVID-19 may be a change in patient expectations for receiving antibiotics. Due to its high prevalence and a lack of available testing, during the first wave of the pandemic, the public was encouraged to presume that ARI symptoms were COVID-19 [26]. Furthermore, messaging from organizations like the Centers for Disease Control (CDC), which campaigned on social media, may have increased awareness among patients of the inappropriateness of antibiotics for viral infections like COVID-19, [27] for which the primary symptoms were cough and sore throat.

A presumption of COVID-19, along with an understanding that antibiotics would not cure COVID-19, could have led to increased tolerance among patients to wait for symptoms to resolve and therefore a temporary decrease in the pressure coming from patients for clinicians to prescribe antibiotics. While few direct comparison studies have been performed, [28] there are indications that fewer patients perceive antibiotics as useful for treating COVID-19 as compared to cold and flu [28–31]. However, as COVID-19 became perceived as more of a concern of the past, there may have been a return to previous prescribing expectations for ARIs. Indeed, while providing fact-checking about antibiotic use for COVID-19 reduces misinformation, the effect has been found to be fleeting [32]. Supporting this hypothesis, antibiotic prescribing for UTI, did not see a similar decline during COVID-19 indicating that the changes in antibiotic prescribing behaviors was not disease independent, nor exclusively a result of a shift to telemedicine. Increased patient antibiotic knowledge has been demonstrated to reduce patient desire for antibiotics and can further reduce antibiotic prescribing beyond clinician-only interventions [33–35]. More research is needed to determine whether a rise in awareness among the public of a widespread viral ARI did lead to reduced patient pressure on prescribers.

Similarly, messaging around COVID-19 and its high incidence in the first pandemic wave, may have created a simplified risk calculation for clinicians. With the default assumption being COVID-19 unless presented with other symptoms indicative of bacterial infection, this may have eased pressure for antibiotic prescribing. It has been demonstrated that when provided with tools to accurately assess the risk of ARI bacterial infection based on patients’ symptoms, clinicians will decrease their antibiotic prescribing rates [36, 37]. However, while still potentially clinically meaningful, [38] rational approaches such as education, feedback, and financial incentives to reduce prescribing have generally only produced 10% reductions in antibiotic prescription rates, whereas studies using alternate approaches such as behavioral economics have reduced prescribing by up to 80% [39–42]. Therefore, future efforts in antibiotic stewardship may benefit from continued study of factors leading to the reduction and rebound in prescribing rates seen in the COVID-19 pandemic, and how those lessons can be translated into antibiotic stewardship interventions.

Additional theories attribute the decline in antibiotic prescribing to a decrease overall in respiratory infections resulting from COVID-19 mitigation measures and consequent disruption to non-COVID-19 respiratory virus activity [5]. However, if prescribing behaviors remained the same, a decrease in non-COVID-19 ARI encounters should have a commensurate decrease in number of antibiotic prescriptions resulting in stable rates of antibiotic prescribing rather than the observed notable decline in antibiotic prescribing rates. The decrease in encounter volume observed in this study (29.4%) is similar to the decrease in outpatient visits observed nationally (30%) [43]. This observed change in prescribing rate, which accounted for change in non-COVID-19 ARI volume, indicates that the observed decline in antibiotic prescriptions are likely a result of something other than a decline in overall non-COVID-19 ARI. Furthermore, the analyses presented in this study do not include COVID-19 ARI visits in the denominator, therefore controlling for the influx of COVID-19 related ARI visits that would artificially decrease prescribing rates (by increasing the denominator size, but not the numerator).

While the volume of ARI encounters did decrease during the pandemic, this alone cannot explain changes in ARI antibiotic prescription rates. The patterns observed between the study sites in this study indicates a potential shift in encounter type resulting from the COVID-19 pandemic. At the onset of the first wave of COVID-19, ARI prescription rates decline most in sites without telemedicine data (NY-B and Utah) and without urgent care (NY-A). As observed in unpublished institutional data from NY-A and Wisconsin, and across institutions in the US, [44] there was a significant increase in the proportion of ARI encounters occurring via telemedicine during the first wave of the COVID-19 pandemic. Therefore, additional telemedicine ARI antibiotic prescriptions may not have been captured within our analyses for the NY-B and Utah health systems. Furthermore, despite a drop in ARI volume, Wisconsin did not observe the same pattern of ARI antibiotic prescription rate drop off observed at the other sites indicating other non-volume factors contributing to antibiotic prescription rates.

This study had several limitations. First, there were variations in how ambulatory care was defined in each health system, and not all visit types were available for all sites (See supplement S1 Table for site specific data characteristics). This limited the ability to directly compare prescribing rates between health systems. However, overall trends in antibiotic prescribing over time were still able to be assessed. Furthermore, the data did not capture all sources of ARI care available outside the study sites (e.g. non-system urgent care). These differences in ambulatory care definitions may explain the limited rebound seen in one health system, which does not include an extensive urgent care network unlike the other study health systems. Additionally, as this study used secondary data and relied on the previous provider documentation of encounter type and antibiotic prescribing based on ICD-10 codes, and did not directly observe each encounter, there is the potential for classification bias if any encounters were misclassified at the time of their occurrence. Second, for simplicity, the same cut points defining the start and end of the first COVID-19 wave were used for all study sites, which may not represent the on the ground reality for each site as the COVID-19 pandemic hit parts of the US before others (e.g. New York City before the Midwest). However, these differences in COVID-19 onset timing are not expected to substantially alter the conclusions of these analyses. Similarly, the analyses did not account for other external factors that were geographic or facilitate specific that may have impacted prescribing behaviors. In particular, differences in access to COVID-19 testing, including home testing, throughout the various phases of the pandemic were not adjusted for, which may have differentially impacted the use of ambulatory visits between the study sites. These variations in study site environment are particularly important to consider when interpreting the aggregate results. Finally, an initial spike in UTI antibiotic prescriptions observed at the first pandemic time point was artificially removed from the trend analyses. The spike was likely observed due to a rapid decline in total UTI ambulatory care visits at the immediate onset of the COVID-19 pandemic. The relatively stable UTI antibiotic prescription rates before and after this timepoint, however, indicates that this spike was likely an aberration and did not represent actual trends in UTI antibiotic prescriptions.

## Conclusions

The decline in antibiotic prescribing for ARI observed after the onset of the COVID-19 pandemic was temporary and does not represent a long-term change in clinician prescribing behaviors. During a period of extreme heightened awareness of a viral cause of ARI a significant and potentially clinically meaningful decrease in clinician antibiotic prescribing was observed. Future efforts in antibiotic stewardship may benefit from continued study of factors leading to this reduction and rebound in prescribing rates.

## Supporting information

S1 Table Study site characteristics and data transformation used. (DOCX)

S2 Table ICD-10 codes for ARI, UTI, and COVID-19, and included antibiotic types. (DOCX)

S3 Table UTI antibiotic prescribing rate trends in the periods of pre-COVID-19, COVID-19 first wave, and post-COVID-19 first wave. (DOCX)

S4 Table ARI antibiotic prescribing rate trends in the periods of pre-COVID-19, COVID-19 first wave, and post-COVID-19 first wave. (DOCX)

S1 Fig COVID-19 first wave change point analysis. (DOCX)

S2 Fig Aggregate ARI antibiotic prescribing trend for all study sites. (DOCX)

S1 File ARI and UTI antibiotic prescribing trends disaggregated by study site. (DOCX)
