# Supplementary material for: Ambulatory antibiotic prescription rates for acute respiratory infection rebound two years after the start of the COVID-19 pandemic
Source: PLoS One. 2024 Jun 25;19(6):e0306195. doi: 10.1371/journal.pone.0306195 (PMC11198751; doi:10.1371/journal.pone.0306195)
Supplement: S1 Table — (DOCX) [file pone.0306195.s001.docx]

**Supplementary Materials**

**Ambulatory antibiotic prescription rates for acute respiratory infection rebound two years after the start of the COVID-19 pandemic**

**Table S1. Study site characteristics and data transformation used**

| **Study Site** | **Encounter Type(s)** | **# Primary care** | **# Urgent care** | **ARI data transformation*** |
| --- | --- | --- | --- | --- |
| **NY-A** | Office Visit, Telemedicine | 121 | 5 | Deseasonalized by multiplicative seasonality |
| **NY-B** | Office Visit | 60 | 51 | Deseasonalized by multiplicative seasonality |
| **Utah** | Office Visit | 22 | 10 | None |
| **Wisconsin** | Office Visit, Telemedicine | 52 | 2 | Deseasonalized by multiplicative seasonality |
| Note: The 2020/04 observation was excluded from URI and UTI models as an outlier due to low visit volume | | | | |
