# Supplementary material for: Ambulatory antibiotic prescription rates for acute respiratory infection rebound two years after the start of the COVID-19 pandemic
Source: PLoS One. 2024 Jun 25;19(6):e0306195. doi: 10.1371/journal.pone.0306195 (PMC11198751; doi:10.1371/journal.pone.0306195)
Supplement: S2 Table — (DOCX) [file pone.0306195.s002.docx]

**Supplementary Materials**

**Ambulatory antibiotic prescription rates for acute respiratory infection rebound two years after the start of the COVID-19 pandemic**

**Table S2 ICD-10 codes for ARI, UTI, and COVID-19, and included antibiotic types**

| **Acute Respiratory Infection** | | **Antibiotics** | | |
| --- | --- | --- | --- | --- |
| B27 | Infectious mononucleosis | amikacin | erythromycin | sulfacetamide |
| B95 | Streptococcus, Staphylococcus, and Enterococcus | amoxicillin | ethambutol | sulfadiazine |
| R04.2 | Hemoptysis | ampicillin | fidaxomicin | sulfamethoxazole |
| R05 | Cough | azithromycin | fosfomycin | tedizolid |
| R06.0 | Dyspnea | aztreonam | gatifloxacin | tetracycline |
| R07.0 | Pain in throat | bacitracin | gentamicin | tigecycline |
| J00 | Acute nasopharyngitis | besifloxacin | imipenim-cilastatin | tobramycin |
| J01 | Acute sinusitis | cefaclor | isioniazid | trimethoprim |
| J02 | Acute pharyngitis | cefadroxil | levofloxacin | vancomycin |
| J03 | Acute tonsillitis | cefazolin | linezolid |  |
| J04 | Acute laryngitis and tracheitis | cefdinir | mafenide |  |
| J05 | Acute obstructive laryngitis and epiglottitis | cefepime | methenamine |  |
| J06 | Acute upper respiratory infections of multiple and unspecified sites | cefiderocol | methionine |  |
| J09 | Influenza due to certain identified influenza viruses | cefixime | metronidazole |  |
| J10 | Influenza due to other identified influenza virus | cefotaxime | minocycline |  |
| J11 | Influenza due to unidentified influenza virus | cefoxitin | moxifloxacin |  |
| J13 | Pneumonia due to Streptococcus pneumoniae | cefpodoxime | mupicrocin |  |
| J14 | Pneumonia due to Hemophilus influenzae | cefprozil | nafcillin |  |
| J15 | Bacterial pneumonia, not elsewhere classified | ceftaroline fosamil | neomycin |  |
| J16 | Pneumonia due to other infectious organisms, not elsewhere classified | ceftazidime | nitrofurantoin |  |
| J17 | Pneumonia in diseases classified elsewhere | ceftizoxime | ofloxacin |  |
| J18 | Pneumonia, unspecified organism | ceftolozane-tazobactam | omadacycline |  |
| J20 | Acute bronchitis | ceftriaxone | oxacillin |  |
| J21 | Acute bronchiolitis | cefuroxime axetil | oritavancin |  |
| J22 | Unspecified acute lower respiratory infection | cephalexin | oxyquinolone |  |
| J36 | Peritonsillar abscess | ciprofloxacin | ozenoxacin |  |
| J39.0 | Retropharyngeal and parapharyngeal abscess | clarithromycin | penicillin |  |
| J39.1 | Other abscess of pharynx | clindamycin | piperacillin-tazobactam |  |
| J40 | Bronchitis | clofazimine | polymyxin |  |
| **Urinary Tract Infection** | | colistin | pyrazinamide |  |
| N30 | Cystitis | dalbavancin | rifabutin |  |
| N39.0 | Urinary tract infection, site not specified | dapsone | rifampin |  |
| R30.0 | Dysuria | daptomycin | rifapentine |  |
| R35.0 | Urinary frequency | delafloxacin | rifaximin |  |
| **COVID-19** | | demeclocycline | sarecycline |  |
| U07.1 | COVID-19 | dicloxacillin | secnidazole |  |
| J12.82 | Pneumonia due to coronavirus disease | doxycycline | silver sulfadiazine |  |
