# Supplementary material for: Ambulatory antibiotic prescription rates for acute respiratory infection rebound two years after the start of the COVID-19 pandemic
Source: PLoS One. 2024 Jun 25;19(6):e0306195. doi: 10.1371/journal.pone.0306195 (PMC11198751; doi:10.1371/journal.pone.0306195)
Supplement: S3 Table — (DOCX) [file pone.0306195.s003.docx]

**Supplementary Materials**

**Ambulatory antibiotic prescription rates for acute respiratory infection rebound two years after the start of the COVID-19 pandemic**

**Table S3. UTI antibiotic prescribing rate trends in the periods of pre-COVID-19, COVID-19 first wave, and post-COVID-19 first wave.**

|  | **Overall** | | **NY-A** | | **NY-B** | | **Utah** | | **Wisconsin** | |
| --- | --- | --- | --- | --- | --- | --- | --- | --- | --- | --- |
|  | **Value** | **95% CI** | **Value** | **95% CI** | **Value** | **95% CI** | **Value** | **95% CI** | **Value** | **95% CI** |
| **Intercept** | 60.1 | 56.72 to 63.47 | 42.71 | 40.53 to 44.9 | 56.73 | 51.63 to 61.83 | 40.31 | 37.87 to 42.76 | 82.77 | 78.52 to 87.01 |
| **Slope Pre-COVID-19** | -0.26 | -0.45 to -0.07 | -0.17 | -0.26 to -0.07 | -0.79 | -1.08 to -0.49 | 0.27 | 0.17 to 0.38 | -0.03 | -0.28 to 0.22 |
| **Step change first wave** | 5.15 | 0.18 to 10.12 | 8.55 | 4.11 to 12.98 | -2.6 | -10.96 to 5.75 | 0.32 | -4.09 to 4.73 | -0.8 | -9.2 to 7.61 |
| **Slope change first wave** | -0.37 | -0.97 to 0.23 | -0.71 | -1.18 to -0.24 | 0.94 | -0.02 to 1.89 | -0.63 | -1.1 to -0.15 | -0.12 | -0.98 to 0.75 |
| **Step change post first wave** | 2.52 | -4.4 to 9.43 | 1.83 | -2.7 to 6.36 | 11.64 | -0.99 to 24.27 | -11.54 | -16.59 to -6.49 | -6.16 | -15.24 to 2.92 |
| **Slope change post first wave** | -0.63 | -1.01 to -0.24 | 0.17 | -0.11 to 0.46 | 1.11 | -0.9 to 3.12 | -0.1 | -0.42 to 0.22 | 0.35 | -0.21 to 0.92 |
