# Supplementary material for: Ambulatory antibiotic prescription rates for acute respiratory infection rebound two years after the start of the COVID-19 pandemic
Source: PLoS One. 2024 Jun 25;19(6):e0306195. doi: 10.1371/journal.pone.0306195 (PMC11198751; doi:10.1371/journal.pone.0306195)
Supplement: S4 Table — (DOCX) [file pone.0306195.s004.docx]

**Supplementary Materials**

**Ambulatory antibiotic prescription rates for acute respiratory infection rebound two years after the start of the COVID-19 pandemic**

**Table S4. ARI antibiotic prescribing rate trends in the periods of pre-COVID-19, COVID-19 first wave, and post-COVID-19 first wave.**

|  | **Overall** | | **NY-A** | | **NY-B** | | **Utah** | | **Wisconsin** | |
| --- | --- | --- | --- | --- | --- | --- | --- | --- | --- | --- |
|  | **Value** | **95% CI** | **Value** | **95% CI** | **Value** | **95% CI** | **Value** | **95% CI** | **Value** | **95% CI** |
| **Intercept** | 54.7 | 51.76 to 57.65 | 44.67 | 42.62 to 46.73 | 54.66 | 49.66 to 59.65 | 37.49 | 31.33 to 43.65 | 69 | 65.36 to 72.63 |
| **Slope Pre-COVID-19** | -0.3 | -0.47 to -0.13 | -0.08 | -0.17 to 0.01 | -1.15 | -1.43 to -0.86 | -0.01 | -0.26 to 0.24 | -0.21 | -0.42 to -0.01 |
| **Step change first wave** | -15.16 | -20.41 to -9.92 | -17.17 | -21.53 to -12.81 | -12.22 | -19.6 to -4.83 | -14.96 | -20.85 to -9.07 | 0.33 | -3.91 to 4.57 |
| **Slope change first wave** | 0.62 | 0.05 to 1.19 | 0 | -0.46 to 0.46 | 1.99 | 1.09 to 2.9 | 0.21 | -0.64 to 1.07 | -0.27 | -0.87 to 0.33 |
| **Step change post first wave** | 0.01 | -6.13 to 6.16 | -10.18 | -14.49 to -5.88 | 25.57 | 13.67 to 37.46 | 1.96 | -9.3 to 13.22 | -3.33 | -10.7 to 4.05 |
| **Slope change post first wave** | 0.03 | -0.32 to 0.39 | 0.23 | -0.04 to 0.51 | 0.55 | -1.24 to 2.34 | -0.31 | -0.93 to 0.31 | 0.68 | 0.26 to 1.09 |
