# Supplementary material for: Ambulatory antibiotic prescription rates for acute respiratory infection rebound two years after the start of the COVID-19 pandemic
Source: PLoS One. 2024 Jun 25;19(6):e0306195. doi: 10.1371/journal.pone.0306195 (PMC11198751; doi:10.1371/journal.pone.0306195)
Supplement: S1 Fig — (DOCX) [file pone.0306195.s005.docx]

**Supplementary Materials**

**Ambulatory antibiotic prescription rates for acute respiratory infection rebound two years after the start of the COVID-19 pandemic**

**Figure S1. COVID-19 first wave Change Point Analysis**

**
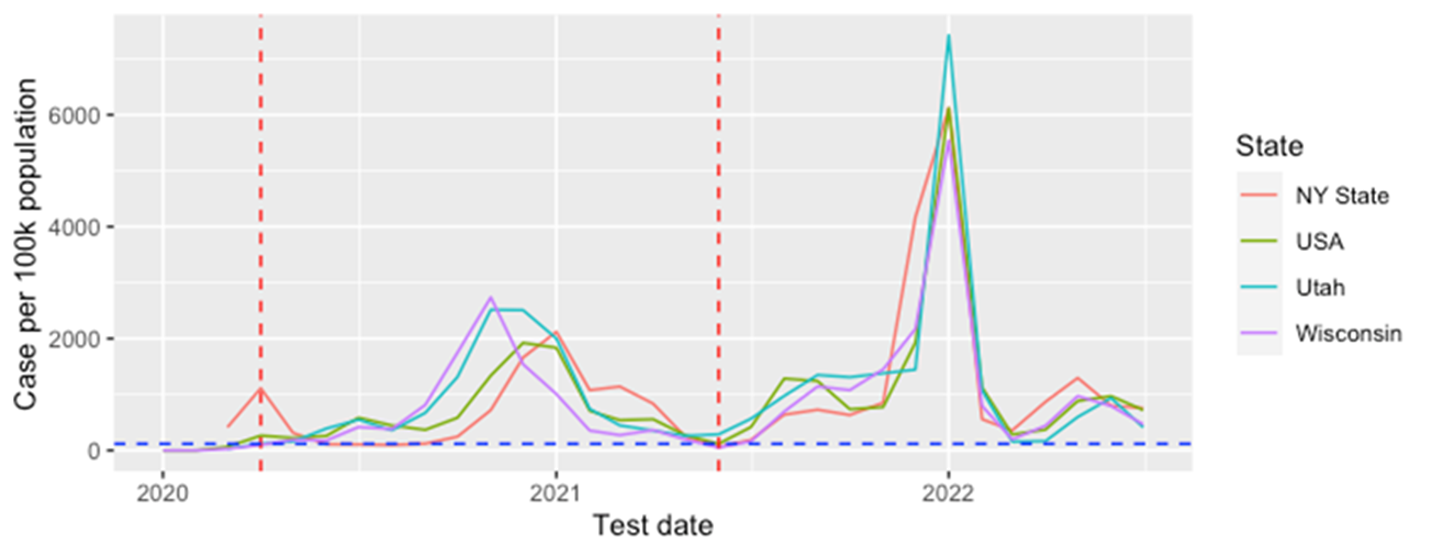
**
