# Supplementary material for: Ambulatory antibiotic prescription rates for acute respiratory infection rebound two years after the start of the COVID-19 pandemic
Source: PLoS One. 2024 Jun 25;19(6):e0306195. doi: 10.1371/journal.pone.0306195 (PMC11198751; doi:10.1371/journal.pone.0306195)

**Supplementary Materials**

**Ambulatory antibiotic prescription rates for acute respiratory infection rebound two years after the start of the COVID-19 pandemic**

**Figure S2. Aggregate ARI antibiotic prescribing trend for all study sites**


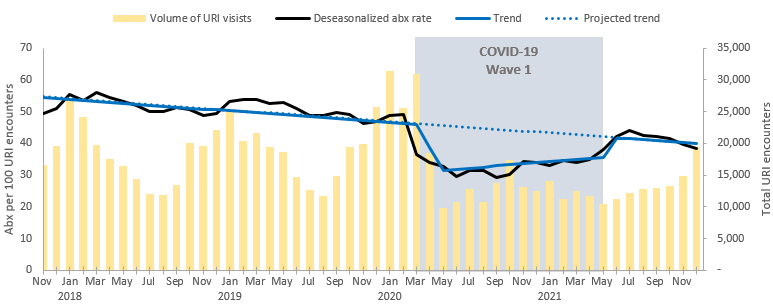

Supplement: S2 Fig — (DOCX) [file pone.0306195.s006.docx]
