# Supplementary material for: Ambulatory antibiotic prescription rates for acute respiratory infection rebound two years after the start of the COVID-19 pandemic
Source: PLoS One. 2024 Jun 25;19(6):e0306195. doi: 10.1371/journal.pone.0306195 (PMC11198751; doi:10.1371/journal.pone.0306195)
Supplement: S1 File — (DOCX) [file pone.0306195.s007.docx]

**Supplementary Materials**

**Ambulatory antibiotic prescription rates for acute respiratory infection rebound two years after the start of the COVID-19 pandemic**

**Figures S3-6. ARI and UTI antibiotic prescribing trends disaggregated by study site**

**S3. NY-A ARI and UTI antibiotic prescribing trends and COVID-19 prevalence, 2017-2022**

**
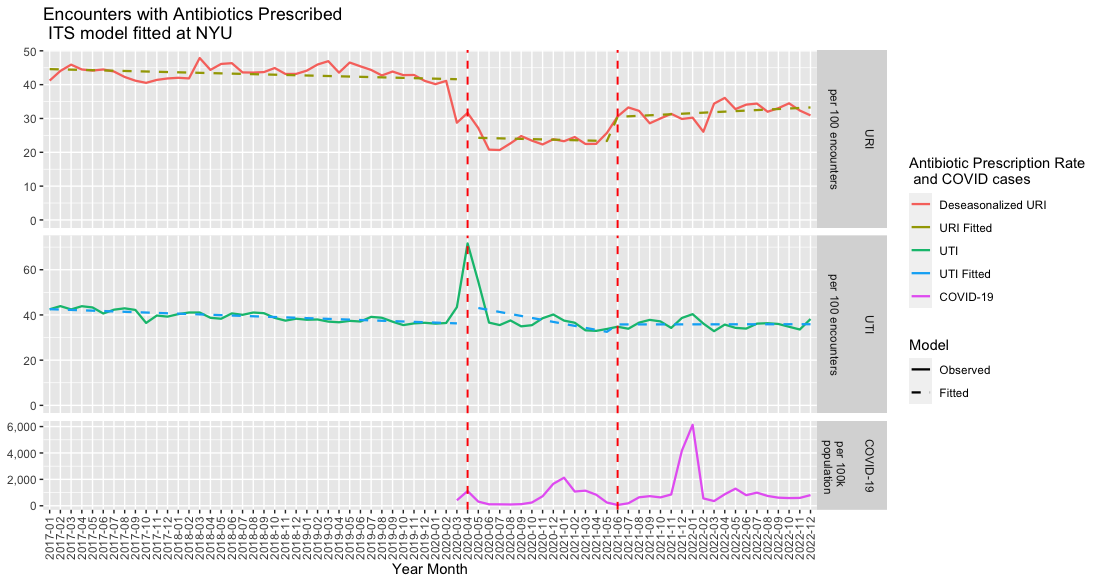
**

**S4. NY-B ARI and UTI antibiotic prescribing trends and COVID-19 prevalence, 2017-2022**

**
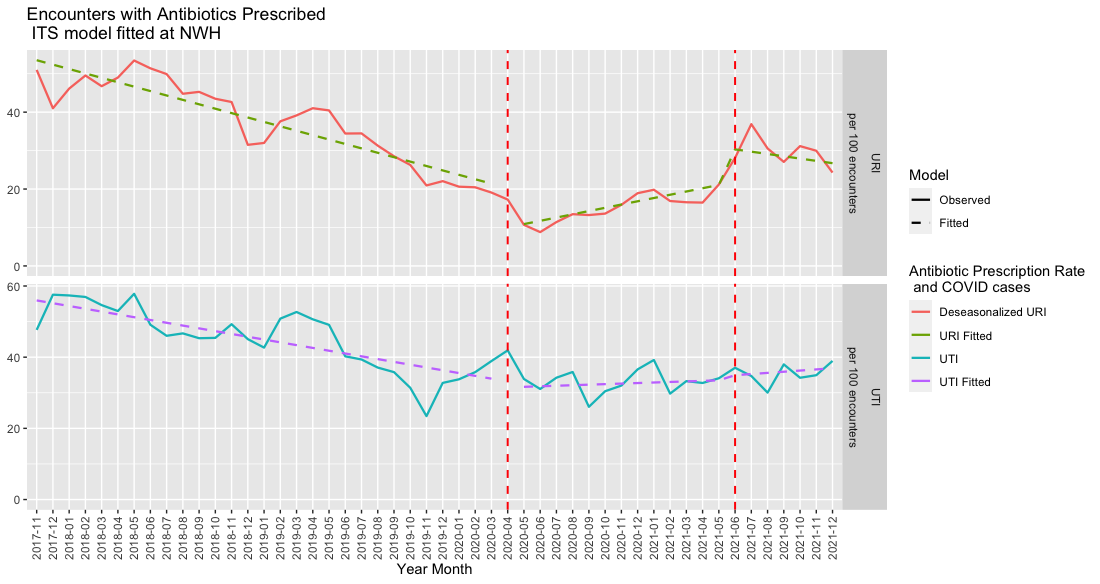
**

**S5. Utah ARI and UTI antibiotic prescribing trends and COVID-19 prevalence, 2017-2022**

**
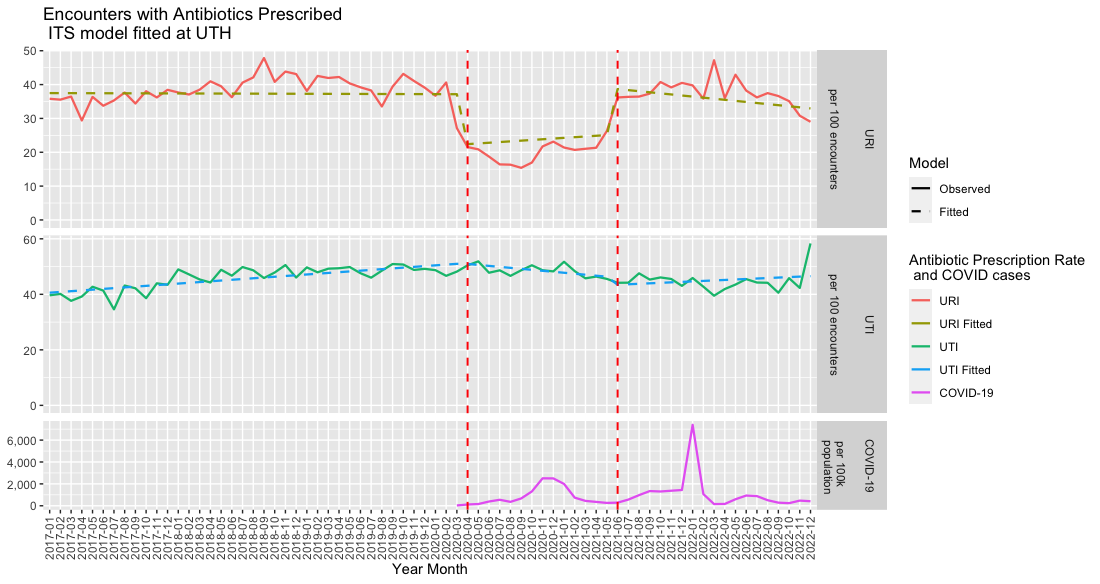
**

**S6. Wisconsin ARI and UTI antibiotic prescribing trends and COVID-19 prevalence, 2017-2022**

**
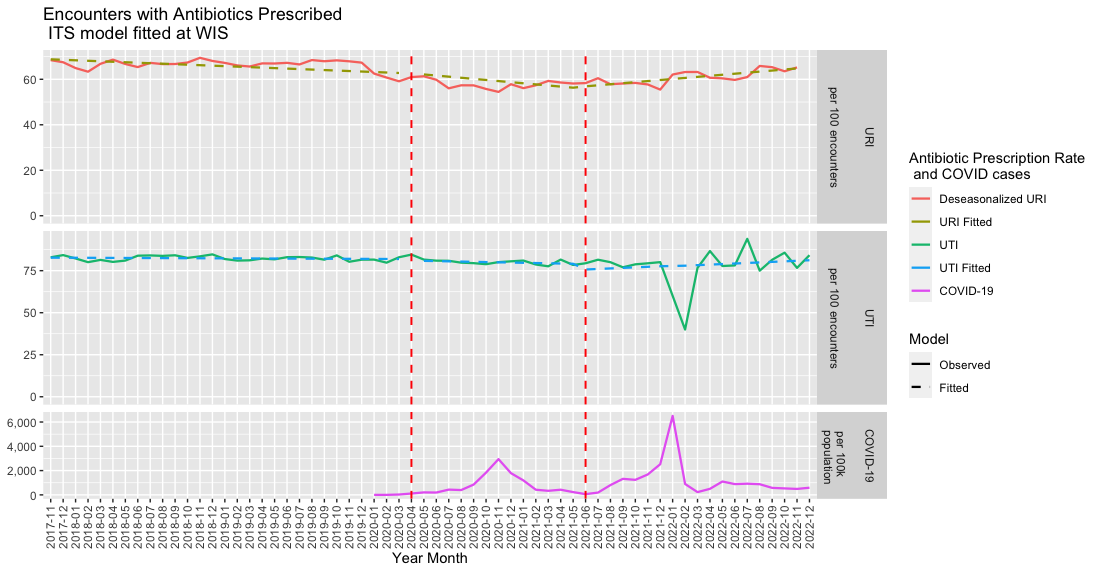
**
